# Chronic Eosinophilic Pneumonia With Overlapping Emphysema and Fibrosis: An Atypical Presentation of Recurrent Pulmonary Tuberculosis

**DOI:** 10.7759/cureus.85228

**Published:** 2025-06-02

**Authors:** Alejandro Cardona, Simón Villa-Pérez, Sofía Cohen-Mejía, Sara Eusse-Ríos, Andrés Felipe Pérez-Quintero, María Angélica Echeverri-Baena

**Affiliations:** 1 Pathology, Hospital Pablo Tobón Uribe, Medellin, COL; 2 School of Medicine, Universidad EIA, Medellin, COL; 3 Internal Medicine, Hospital Pablo Tobón Uribe, Medellin, COL

**Keywords:** atypical presentation, emphysema, eosinophilic pneumonia, pulmonary fibrosis, tuberculosis

## Abstract

This case report describes an atypical presentation of recurrent pulmonary tuberculosis in a patient with chronic respiratory symptoms and eosinophilia, initially misdiagnosed as chronic eosinophilic pneumonia. Despite suggestive clinical and radiological features, a final diagnosis was only reached after prolonged microbiological culture. The case underscores the diagnostic complexity of interstitial lung diseases and emphasizes the importance of maintaining a broad differential, particularly in tuberculosis-endemic regions.

## Introduction

Eosinophils are myeloid-derived leukocytes first identified by Paul Ehrlich in 1879. Under physiological conditions, eosinophils are absent from the airways and lung parenchyma; their recruitment and infiltration into tissues are mediated by T-helper type 2 (Th2) lymphocytes and the secretion of multiple cytokines, including interleukin-3 (IL-3), IL-5, IL-13, and granulocyte-macrophage colony-stimulating factor (GM-CSF) [1].

Eosinophilic lung diseases (ELD) comprise a heterogeneous group of disorders characterized by eosinophil-mediated pulmonary involvement [2,3]. This group encompasses idiopathic pulmonary entities such as acute and chronic eosinophilic pneumonia, systemic conditions like eosinophilic granulomatosis with polyangiitis and idiopathic hypereosinophilic syndrome, as well as diseases of known etiology, including allergic bronchopulmonary aspergillosis, parasitic infections, and drug-induced lung injury [3-5]. A universally accepted classification of ELDs is lacking; however, they are often categorized based on their temporality, underlying cause, or the anatomical structures involved [5].

The diagnostic evaluation of eosinophilic pneumonia mandates a thorough exclusion of primary causes, including drug exposures and infections [6,7]. Although pulmonary tuberculosis has been reported as a rare cause of peripheral eosinophilia, its clinical, radiological, and bronchoalveolar lavage features usually allow differentiation from idiopathic eosinophilic pneumonia [8].

In this paper, we report a case of secondary eosinophilic pneumonia manifesting as an atypical presentation of pulmonary tuberculosis.

## Case presentation

A 60-year-old woman from Medellín, Colombia, with a history of active smoking (23.5 pack-years) and previously treated pulmonary tuberculosis three years prior, presented with a two-year history of intermittent dyspnea and productive cough with mucoid sputum, which partially improved with acetaminophen. She experienced an acute exacerbation of symptoms, leading to her admission through the emergency department. On physical examination, auscultation revealed bilateral velcro-like crackles at the bases and wheezing at the right apex.

Laboratory evaluation demonstrated marked peripheral eosinophilia (2,875 cells/mm³) and an elevated C-reactive protein level (22.5 mg/dL). Polymerase chain reaction (PCR) assays for SARS-CoV-2, sputum PCR for *Mycobacterium tuberculosis*, and HIV testing were all negative.

**Figure 1:**
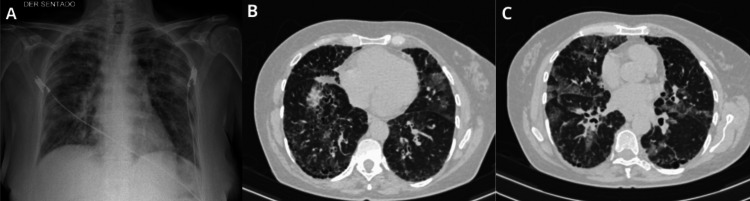
Diagnostic imaging. (A) AP chest radiograph showing bilateral interstitial opacities. (B, C) Chest CT images demonstrating bilateral ground-glass opacities with random central and peripheral distribution, parenchymal hyperdensities with air bronchograms in the right apex, and subpleural lesions in the left lung.

Non-contrast chest CT revealed centrilobular emphysema predominantly at the apices, basal fibrotic changes with reticular opacities, traction bronchiectasis, areas of honeycombing, and diffuse bilateral ground-glass opacities with a random peripheral distribution. Additionally, small focal areas of consolidation were noted in the right upper lobe (Figure 1). Pulmonary function testing showed a severe restrictive ventilatory defect and moderately reduced diffusion capacity for carbon monoxide (DLCO).

A diagnostic bronchoscopy with bronchoalveolar lavage (BAL) revealed 25% eosinophils on cytology, with initial negative microbiological workup, including aerobic cultures, Gram staining, potassium hydroxide (KOH) mount, methenamine silver staining, galactomannan assay, pneumonia multiplex PCR, *Pneumocystis jirovecii* PCR, and *Mycobacterium tuberculosis* PCR.

Given these findings, a diagnosis of chronic eosinophilic pneumonia superimposed on emphysematous and fibrotic lung disease was considered, and corticosteroid therapy with prednisolone 50 mg daily was initiated with close outpatient monitoring.

Subsequently, after a 39-day incubation period, culture of the BAL fluid yielded acid-fast bacilli belonging to the *Mycobacterium tuberculosis* complex. The patient was re-evaluated, and anti-tuberculous therapy with the standard HRZE quadruple regimen was initiated.

## Discussion

ELDs comprise a heterogeneous group of pulmonary disorders characterized by infiltration of eosinophils into the interstitium and alveolar spaces. Under normal conditions, eosinophils account for less than 2% of cells in BAL fluid; however, in ELD, levels may exceed 25% [2,3,9,10]. Among these disorders, acute and chronic eosinophilic pneumonia (CEP) are the primary idiopathic entities confined to the lungs.

CEP is an uncommon, slowly progressive disease of unknown etiology. Diagnosis is frequently delayed due to its nonspecific and relatively mild symptomatology, which often overlaps radiologically with other conditions such as organizing pneumonia [5].

CEP accounts for less than 3% of all interstitial lung diseases. Epidemiological data are limited, but a small retrospective study estimated an incidence of 0.23 cases per 100,000 inhabitants annually [2]. Nonetheless, it is the most common form of eosinophilic pneumonia in non-tropical regions where parasitic infections are rare [4]. CEP predominantly affects women (2:1 female-to-male ratio), with a mean age at diagnosis of approximately 50 years, although it may occur at any age. Unlike acute eosinophilic pneumonia, most CEP patients are non-smokers; 65% have a history of bronchial asthma, and 50% present with allergic comorbidities such as allergic rhinitis, atopic dermatitis, nasal polyposis, or urticaria. Prior breast cancer radiation therapy has also been identified as a predisposing factor. The clinical course is usually subacute to chronic, with symptoms evolving over several weeks or months before diagnosis [2,4,6].

CEP diagnosis requires the presence of respiratory symptoms persisting for more than two to four weeks, bilateral patchy consolidations or ground-glass opacities predominantly in the upper lobes and subpleural areas (classically described as a *photographic negative* of pulmonary edema), and peripheral eosinophilia (>1,000 cells/mm³) and/or BAL eosinophilia exceeding 40%. Other imaging features may include septal thickening, nodules, pleural effusion, or the *atoll* (reverse halo) sign. Laboratory findings often demonstrate peripheral eosinophilia (66%-95%) and elevated IgE levels in up to 50% of patients. Pulmonary function testing reveals variable patterns, including normal, restrictive, or obstructive profiles, with a consistent reduction in DLCO (Table 1) [6,11].

**Table 1:** Key histological characteristics of eosinophilic pneumonias. Adapted from [11].

Acute eosinophilic pneumonia with respiratory failure	Idiopathic chronic eosinophilic pneumonia
Diffuse alveolar damage with a marked eosinophilic infiltration	Focal areas of alveolar consolidation predominantly in the peripheral lung fields; prominent aggregates of lymphocytes and eosinophils within alveolar septa and spaces; frequent interstitial fibrosis and organizing pneumonia

The pathogenesis of CEP remains unclear but involves excessive eosinophil recruitment into the pulmonary interstitium [5]. BAL fluid from patients typically shows elevated type 2 cytokines, highlighting their role in eosinophil activation and accumulation. Acute eosinophilic pneumonia exhibits higher cytokine levels compared to chronic forms, although eosinophil counts are similar in both [1,7,12,13]. IL-5 plays a pivotal role, promoting eosinophil recruitment and inhibiting apoptosis. This cytokine is secreted by activated Th2 lymphocytes, which are themselves recruited by chemokines such as RANTES (Regulated on Activation, Normal T Expressed and Secreted). Other important mediators include IL-6, IL-10, and eotaxin [5].

Given the substantial overlap between primary and secondary causes of pulmonary eosinophilia, an extensive etiological investigation is imperative at initial evaluation [4]. Although many cases are idiopathic, proposed triggers include acute hypersensitivity reactions to inhaled antigens such as tobacco smoke or chemical vapors [2]. The differential diagnosis encompasses infections (e.g., tuberculosis, brucellosis, psittacosis, coccidioidomycosis, histoplasmosis, pneumocystosis, and parasitic infections), drug reactions, autoimmune diseases, and malignancies (Table 2) [7].

**Table 2:** Etiological spectrum of eosinophilic pneumonia. Adapted from [12].

Etiology		Examples
Known cause		Medications, parasites, radiation/toxics, fungi, or mycobacterium
Diseases that can lead to pulmonary eosinophilia	Diffuse Lung Diseases	Cryptogenic organizing pneumonia, Hypersensitivity pneumonitis, idiopathic pulmonary fibrosis, Langerhans cell histiocytosis, sarcoidosis
	Malignancies	Leukemia, lymphoma, lung cancer, adenocarcinoma, or squamous cell carcinoma with metastasis
	Connective tissue diseases	Rheumatoid arthritis, Sjögren's syndrome
	Other	Asthma, eosinophilic bronchitis, organizing pneumonia, Heiner syndrome, lung transplant, SARS-CoV-2 infection, acute eosinophilic pneumonia after COVID-19 mRNA vaccination

The clinical presentation of CEP varies, depending on whether it is isolated to the lungs or associated with systemic diseases, such as hypereosinophilic syndromes or infections. Most cases exhibit a gradual, subacute onset. Dyspnea and cough are present in 60%-90% of patients, while rhinitis, sinusitis (20%), and hemoptysis (10%) are less common. Physical examination may reveal wheezing or fine crackles; systemic symptoms such as fever, anorexia, and weight loss may also occur. Respiratory failure is rare in idiopathic CEP [4,12,13].

CEP is diagnosed by combining persistent respiratory symptoms (>2 weeks), alveolar eosinophilia (>25%, ideally >40%) or marked peripheral eosinophilia (>1,000-1,500 eosinophils/mm³), bilateral peripheral alveolar opacities, and exclusion of secondary causes [1,3]. Imaging typically demonstrates the *photographic negative* pattern of pulmonary edema, but septal thickening, reversed halo signs, nodules, and bronchial thickening can also be observed. Up to 50% of patients exhibit elevated IgE. Pulmonary function testing frequently reveals a decreased DLCO, with obstructive, restrictive, or mixed patterns possible.

Systemic corticosteroid therapy remains the standard treatment, with an excellent initial response rate. Although no standardized regimen exists, prednisolone 0.5 mg/kg/day for two weeks followed by gradual tapering over six to twelve months is commonly recommended. Inhaled corticosteroids may help prevent relapses, which occur in up to 50% of patients after tapering [3,5]. Spontaneous remission occurs in approximately 10% of cases [5].

Tuberculosis, in contrast, is an infectious disease caused by *Mycobacterium tuberculosis*, ranking among the leading causes of morbidity and mortality worldwide. Although primarily a pulmonary pathogen, it can affect virtually any organ system, known as extrapulmonary tuberculosis, and organs like the kidneys, bones, intestines, liver, skin, pleura, meninges, among others [14,15].

*Mycobacterium tuberculosis* initially targets alveolar macrophages, evading phagolysosomal degradation and establishing an intracellular niche that promotes granuloma formation. This facilitates latent infection but may progress to active disease, with clinical manifestations such as chronic cough, fever, weight loss, and night sweats [16-18].

Although traditionally considered a macrophage and T cell-mediated infection, recent studies reveal a role for eosinophils in the host immune response to tuberculosis. Bohrer et al. demonstrated that eosinophils accumulate in pulmonary granulomas and contribute to bacterial control and host survival in experimental models [19]. Further work by Bohrer et al. elucidated that eosinophil recruitment occurs early via GPR183 receptor signaling, suggesting a protective role during the initial stages of infection [20].

Accordingly, tuberculosis may present atypically as eosinophilic pneumonia, as previously documented by Vijayan et al., who reported cases of confirmed pulmonary tuberculosis associated with eosinophilic pneumonia, resolving with anti-tuberculous therapy [8].

## Conclusions

This case emphasizes the complexity of diagnosing pulmonary conditions with overlapping clinical and radiologic features. It demonstrates how tuberculosis can present atypically, mimicking chronic eosinophilic pneumonia, and reinforces the need to maintain a broad differential diagnosis. The presence of eosinophilia, emphysema, fibrosis, and ground-glass opacities initially suggested a non-infectious etiology, but further investigation revealed an infectious cause. This highlights the importance of comprehensive microbiological testing, particularly in regions where tuberculosis is endemic. The case also raises important questions about the role of eosinophils in pulmonary infections and the challenges of managing coexisting conditions that require distinct therapeutic strategies. A multidisciplinary approach and early identification of infectious agents are essential to ensure accurate diagnosis and appropriate treatment.
